# Attenuation of an adult T-cell leukemia skin lesion after treatment of a concomitant herpes simplex infection: a case study

**DOI:** 10.1186/1743-422X-9-224

**Published:** 2012-10-01

**Authors:** Hajime Tomita, Fumihide Ogawa, Sayaka Kuwatsuka, Fumi Toriyama, Shinichirou Yasumoto, Shimeru Kamihira, Atsushi Utani

**Affiliations:** 1 Department of Dermatology, Nagasaki University Graduate School of Biomedical Sciences, 1-7-1 Sakamoto, Nagasaki, 852-8501, Japan; 2 Department of Dermatology, Japanese Red Cross Nagasaki Genbaku Hospital, Nagasaki, Japan; 3 Department of Dermatology, Kurume University School of Medicine, Kurume, Japan; 4 Department of Laboratory Medicine, Nagasaki University Graduate School of Biomedical Sciences, Nagasaki, Japan

**Keywords:** Adult T-cell leukemia virus type 1, Herpes simplex, Eczema herpeticum

## Abstract

We report the development and treatment of eczema herpeticum in a 51-year-old male suffering from adult T-cell leukemia (ATL). Lesions of eczema herpeticum coexisted with the skin lesions of ATL. Treatment of eczema herpeticum resulted in a concomitant improvement in the symptoms of ATL, including a reduction in the size of the ATL plaques, for over 2 months before relapse.

## Background

Eczema herpeticum is an uncommon viral infection caused by the herpes simplex virus (HSV). It affects patients with a pre-existing primary dermatological condition, such as atopic dermatitis, psoriasis, mycosis fungoides, and/or burns [1]. Although HSV infections occur frequently in adult T-cell leukemia (ATL) patients, eczema herpeticum at ATL skin lesions has never been reported before in the literature. In this Case Report, we describe the occurrence and treatment of eczema herpeticum in a patient suffering from ATL.

## Case presentation

**Figure 1:**
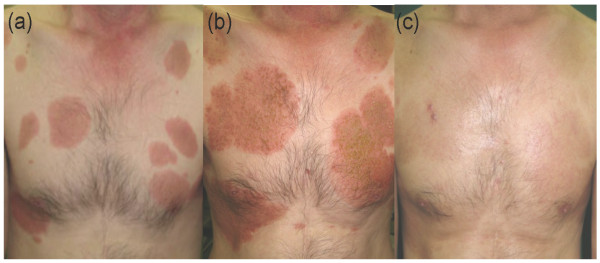
**Clinical pictures of theanterior trunk.** Timings of the clinical pictures are depicted in Figure 4. (**a**) ATL skin lesions on the chest 2 months before the onset of HSV infection. (**b**) Small vesicles, crusts, and erosion developing on the pre-existing ATL skin lesions. (**c**) Complete disappearance of the ATL skin lesions as well as eczema herpeticum improving in parallel 14 days after treatment.

**Figure 2:**
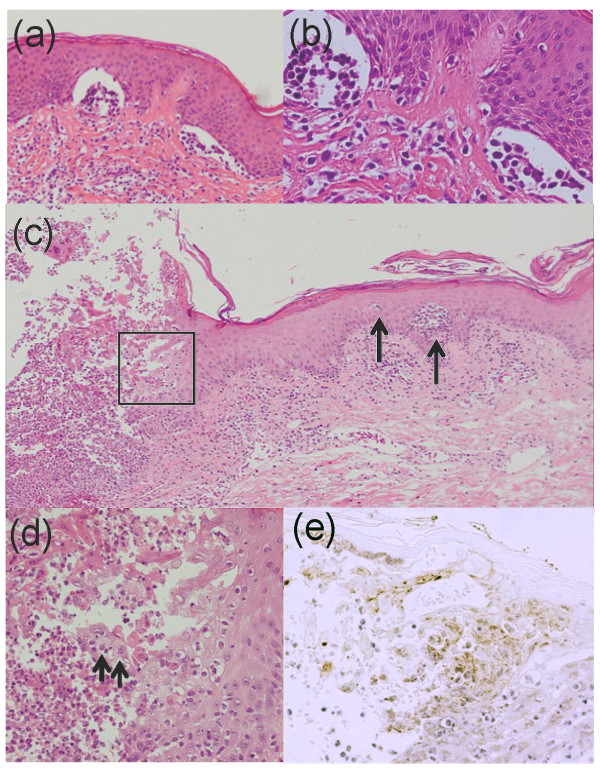
**Histopathological examinations.** Time course of the biopsies is shown in Figure 4. (**a**) (**b**) Biopsy from erythema on the chest showed clustering atypical lymphocytes at the dermo-epithelial junction, also known as Pautrier’s microabscesses and a moderate infiltration of atypical cells in the upper dermis. (hematoxylin and eosin (H&E) stain, original magnification (**a**): ×100; (**b**): ×400). (**c**) Biopsy of the vesicles demonstrated erosion with the degenerated epidermis and concomitantly clustering atypical lymphocytes, Pautrier’s microabscesses (arrows) (H&E stain, original magnification × 40). (**d**) Magnified view of the square in (**c**) revealed acantholytic and swollen cells (arrows) with neutrophil (H&E stain, original magnification × 400). (**e**) HSV in epidermal cells at vesicles (Immunohistochemical staining with anti-HSV antibody, original magnification × 400).

**Figure 3:**
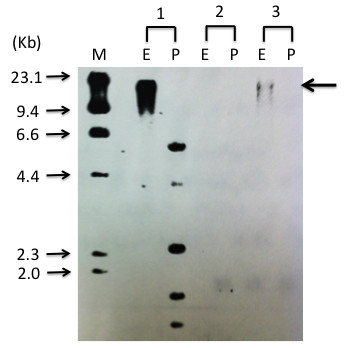
**Southern blot analysis ofDNA from the ATLskin lesion on theanterior chest.** Lane M: molecular weight marker, lane 1, samples of HTLV-1 monoclonal integration; 2, samples from normal volunteer; 3, samples from the patient’s skin lesion. E, EcoR1-digested sample; P, PstI-digested sample. The arrow indicates a monoclonal HTLV-1 proviral integration.

A 51-year-old Japanese male with an 11-year history of ATL presented with erythematous plaques all over his torso (Figure 1a). A test for serum anti-human T-cell leukemia virus type 1 (HTLV-1) antibody was positive. Histological examination of a biopsy specimen taken from an infiltrated erythema on the chest showed clustered atypical lymphocytes at dermo-epithelial junction, also known as Pautrier’s microabscesses, and infiltration of atypical cells in the upper dermis (Figure 2a and b). Simultaneously, southern blot analysis of DNA from an ATL skin lesion demonstrated a faint but obvious signal in the lane containing the EcoRI-digested sample (Figure 3; see arrow), although there was no signal in the lane containing the PstI-digested sample, probably due to the low amount of sample (Figure 3). These results indicate monoclonal integration of proviral HTLV-1 in the skin lesion. Laboratory examinations revealed normal levels of serum calcium, normal levels of lactate dehydrogenase (LDH), and no abnormal cells. A computed tomography (CT) scan showed an absence of visceral involvement, including no lymph node swelling. The patient was therefore diagnosed with indolent ATL. He had been undergoing treatment with a combination of psoralen-ultraviolet A therapy, narrow-band ultraviolet B radiation, and topical steroid ointment for 10 years at our hospital. However, the lesions had gradually increased and become enlarged over this period.

Small vesicles, crusts, erosion, and infiltrated itchy erythemas suddenly developed in the almost every pre-existing ATL skin lesions on the entire trunk (Figure 1b). Examination of a biopsy specimen revealed acantholysis and ballooning degeneration of epidermal keratinocytes in the vesicles (Figure 2c and d). These degenerated epidermal cells were positive for an anti-HSV antibody that recognized both HSV-1 and HSV-2 (Figure 2e). In addition, Pautrier’s microabscesses were observed adjacent to the erosion in the biopsied sample (Figure 2c). An enzyme immunoassay revealed levels of serum immunoglobulin G and M antibodies against HSV of over 128 (normal range <2.0) and 0.77 (normal range <0.8), respectively. These results indicated a recurrent HSV infection that was strictly limited to the pre-existing ATL skin lesions.

**Figure 4:**
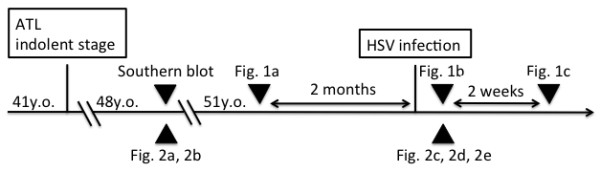
**Time course of clinicalevents and examinations.**

Acyclovir was administered intravenously in a daily dose of 750 mg for 7 days. Within 7 days, re-epithelialization of the erosion was achieved, and the ATL skin lesions and eczema herpeticum improved concomitantly (Figure 1c). The clinical response of the ATL lesions to the anti-herpes treatment persisted for more than 2 months; however, relapse then occurred, with lesions gradually reappearing at locations that were different from those of the primary lesions. The course of clinical episodes and examinations in this case is depicted in Figure 4.

## Discussion

Development of eczema herpeticum appears to be linked to defects in cellular immunity and disruption of the skin barrier. A report suggested that the number of regulatory T (Treg) cells increases in ATL patients [2]. Treg cells hamper the development of immunity against viral infections, such as HSV [3,4]. In addition, HTLV-1 activates cellular genes, including the cytokine gene IL-4 [5]. High levels of IL-4 allow the development of eczema herpeticum by suppressing the expression of cathelicidin peptide LL-37, which exhibits activity against HSV [6]. It is also noteworthy that IL-17 mRNA is highly expressed in HTLV-1-infected T-cells [7] and that IL-17-mediated inhibition of natural killer cell activity induces eczema vaccinatum in mice [8]. Together with disruption of the skin barrier in the ATL lesions, UV-irradiation and topical steroid treatment may also participate in HSV infection. Furthermore, increased Treg cells and high levels of IL-4 and/or IL-17 associated with ATL infection may lead skin conditions vulnerable to HSV infection.

A study has shown that adenovirus vaccine injection into the tumoral lesions of cutaneous T-cell lymphoma leads to tumor reduction by the induction of interferon gamma, a subtype 1 helper T (Th1) cell cytokine [9]. In the present case, the Th1-dominant immune response for anti-viral immunity [10] may have been activated following HSV infection, whereupon it contributed to the attenuation of the ATL skin lesions.

## Conclusions

HSV infection affected our patient only within the confines of pre-existing ATL skin lesions on his torso. Interestingly, these ATL skin lesions improved in parallel with the symptoms of HSV infection upon treatment with acyclovir. This uncommon phenomenon observed in the present case may shed a light to a development of novel strategies for treatment of ATL.

## Consent

Written informed consent was obtained from the patient for publication of this Case Report and any accompanying images. A copy of the written consent is available for review by the Editor-in-Chief of this journal.

## Abbreviations

ATL: Adult T-cell leukemia; CT: Computed tomography; H&E: Hematoxylin and eosin; HSV: Herpes simplex virus; HTLV-1: Human T-cell leukemia virus type 1; LDH: Lactate dehydrogenase; Th1: Subtype 1 helper T; Treg: Regulatory T.

## Competing interests

The authors declare that they have no competing interests.

## Authors’ contributions

HT wrote and edited the manuscript. HT, FK, and FT carried out collection of patient data and patient monitoring through the whole period of follow up. FO and AT edited the manuscript. SY and SK participated in the study design and coordination and helped to draft the manuscript. All authors read and approved the final manuscript.
